# The utility of intravenous ketamine for the management of intraoperative penile erection: a retrospective single-center analysis of endourological surgeries over a 4-year

**DOI:** 10.1186/s12894-020-0574-1

**Published:** 2020-01-28

**Authors:** Aşkın Eroğlu, Bahattin Tuncalı, Rahmi Gökhan Ekin

**Affiliations:** 1 Department of Urology, Başkent University Research and Training Medical Center, Izmir, Turkey; 2 Department of Anesthesia and Reanimation, Başkent University Research and Training Medical Center, Izmir, Turkey; 3 Department of Urology, Urla State Hospital, Izmir, Turkey

**Keywords:** Intraoperative penile erection ∙ ketamine ∙ endourology ∙ treatment outcome

## Abstract

**Background:**

To assess the prevalence of intraoperative penile erection in our endourology practice and the utility of intravenous ketamine in the management of the condition.

**Methods:**

Of 402 endoscopic urological procedures performed in our clinic over a 4-year (2015–2019) period, a total of 9 cases with intraoperative penile erection impeding instrumentation during endourological surgery were included. Data on patient age, weight, height, American Society of Anesthesiologists (ASA) physical status classification system scores, type and duration of surgery, type and level of anesthesia, onset of erection, treatment characteristics and treatment outcome were recorded for each patient.

**Results:**

The mean (SD) age was 68.3 years (range, 66.0–77.0 years). ASA physical status category I and II were noted in 55.6 and 44.4% of patients, respectively. All cases received spinal anesthesia (*n* = 9) at T8–10 dermatome levels, for TURP in 7 (77.8%) cases and for TURBT in 2 (22.2%) cases. The onset of penile erection was post-urethroscope in 7 (77.8%) cases. The average total ketamine dose was 34.3 mg (range, 18.0–75.0 mg). The average duration of the operation was 91.7 min (range, 40.0–140.0 min). Ketamine treatment resulted in resolved erection with delayed procedure in 7 (77.8%) cases, while conversion to general anesthesia was required in 2 (22.5%) cases.

**Conclusions:**

In conclusion, the prevalence of intraoperative penile erection during spinal anesthesia for endourological surgery was 2.2% in our experience. These findings demonstrated that intravenous injection of ketamine is an effective and safe method for immediate resolution of intraoperative penile erection with a high success rate.

## Background

Penile tumescence resulting in partial or total erection under anesthesia at the time of endoscopic urological surgery is a relatively infrequent but challenging condition with reported incidence of 0.1 to 2.4% [1–4]. While the etiology is unclear, it generally follows the local stimulation of the penis during skin preparation or instrumentation [3, 5]. Intraoperative penile erection may lead to delay or cancellation of the surgery by impeding urological instrumentation or safely reaching the prostate or bladder together with the greater risk of complications such as bleeding, urethral trauma and stricture formation [2, 4, 6].

To date, many pharmacological treatments to produce detumescence have been proposed, such as intracavernous injection of ephedrine, phenylephrine, metaraminol, noradrenaline and adrenaline [4, 7–10] or intravenous injection of ketamine [6, 11–13], dexmedetomidine [5], terbutaline [14] and glycopyrrolate [15].

However, each of these has shown varying levels of efficacy and safety with no consensus a fully effective treatment protocol without side-effects [4–6].

The aim of this paper was to present the prevalence of intraoperative penile erection in our endourological surgery practice and the utility of intravenous ketamine in the management of the condition.

## Methods

### Study population

Of 402 endoscopic urological procedures performed in our clinic over a 4-year (2015–2019) period, a total of 9 cases with intraoperative penile erection development impeding instrumentation during endourological surgery were included in this retrospective study.

Written informed consent was obtained from each patient following a detailed explanation of the objectives and protocol of the study which was conducted in accordance with the ethical principles stated in the “Declaration of Helsinki” and was approved by the Institutional Ethics Committee.

### Assessments

Data on patient age, weight, height, American Society of Anesthesiologists (ASA) physical status classification system scores, type and duration of surgery, type and level of anesthesia, onset of erection, treatment characteristics and treatment outcome were recorded for each patient.

### Management of intraoperative penile erection

Intravenous ketamine treatment, titrated to a total dose of up to 75 mg in accordance with Ramsay sedation score of 3, was administered to resolve penile tumescence and rigid erection, which prevented performance of surgery. All patients also received midazolam (0.02 mg/kg) and fentanyl (1 μg/kg) to prevent ketamine-related complications. The surgical procedure was restarted in those with a successfully resolved rigid erection.

### Statistical analysis

Statistical analysis was made using computer software (SPSS version 22.0, IBM, NY,USA). Descriptive statistics were stated as mean ± standard deviation (SD), median, IQR (Inter Quartil Range) and percentage (%), where appropriate.

## Results

### Overall endourological surgery population

The characteristics of the overall endourological surgery population (*n* = 402) are presented in Table 1. The mean age was 69.1 ± 10.2 years. TURP (38.1%) and TURBT (28.4%) were the most common operations. Spinal anesthesia was applied to the majority of patients (72.9%) and the average duration of surgery was 53.4 min. Intraoperative penile erection developed in 9 (2.2%) patients (Table 1).

**Table 1:** Characteristics of the overall endourological surgery population (*n* = 402)

Overall patient characteristics (*n* = 402)		
Age (years), mean (SD)		69.1 (10.2)
Median (IQR)		69 (13)
Weight (kg)		78.4 (11.3)
		78 (15)
Height (cm)		171.9 (5.9)
		172 (8)
ASA physical status category, n (%)	I	151 (37.6)
	II	239 (59.5)
	III	12 (2.9)
Comorbidities, n (%)		
Hypertension		174 (43.3)
Type 2 diabetes mellitus		81 (20.1)
Coronary artery disease		47 (11.7)
Arrhythmia		48 (11.9)
Chronic obstructive pulmonary disease		36 (9.0)
Chronic renal failure		5 (1.2)
Thyroid disease		8 (2.0)
Obstructive sleep apnea syndrome		2 (0.5)
Ankylosing spondylitis		
	TURP	153 (38.1)
Type of surgery, n(%)	TURBT	114 (28.4)
	Cystoscopy	84 (20.9)
	Internal urethrotomy	8 (2.0)
	Ureteroscopy	2 (0.5)
	Cystolithotomy	7 (1.7)
	Endoscopic ureter stone	14 (3.5)
	TUR biopsy	20 (5)
Duration of surgery (min), mean (SD) 53.4 (26.0)		53.4 (26.0)
Type of anesthesia, n (%)	Spinal	293 (72.9)
	General anesthesia	109 (27.1)
Intraoperative penile erection,n(%)	Yes	9 (2.2)
	No	393 (97.8)

*ASA* American Society of Anesthesiologists, *TURP* Transurethral resection of the prostate, *TURBT* Transurethral resection of bladder tumor

### Cases with intraoperative penile erection

The individual and total characteristics of cases with intraoperative penile erection (*n* = 9) are presented in Table 2.

**Table 2:** Cases with intraoperative penile erection (*n* = 9)

Case	ASA	Typeof surgery	Duration of surgery	Anesthesia/Level	Onset of erection	Treatment	Outcome
1	1	TURP	55	Spinal/T10	Post-urethroscope	Ketamine 75 mg	Conversion to GA
2	1	TURP	90	Spinal/T8	Post-urethroscope	Ketamine 25 mg	Delayed procedure
3	2	TURBT	130	Spinal/T8	Post-urethroscope	Ketamine 50 mg	Conversion to GA
4	1	TURP	140	Spinal/T10	Pre-urethroscope	Ketamine 25 mg	Delayed procedure
5	2	TURBT	40	Spinal/T10	Post-urethroscope	Ketamine 20 mg	Delayed procedure
6	2	TURP	90	Spinal/T8	Post-urethroscope	Ketamine 21 mg	Delayed procedure
7	1	TURP	95	Spinal/T8	Post-urethroscope	Ketamine 18 mg	Delayed procedure
8	1	TURP	90	Spinal/T10	Pre-urethroscope	Ketamine 25 mg	Delayed procedure
9	2	TURP	95	Spinal/T8	Post-urethroscope	Ketamine 50 mg	Delayed procedure
Total (*n* = 9)							
Age (years)	Mean (SD)		68.3 (6.6)				
Weight (kg), mean (SD)	Median (IQR)		69.0 (8)				
Median (IQR)			80.7 (7.8)				
Height (cm), mean (SD)			82 (6)				
Median (IQR)		172 (5)	174.6 (7.7)				
ASA physical status							
category, n (%)	I		5 (55.6)				
Type of operation, n (%)	II		4 (44.4)				
	TURP		7 (77.8)				
Type of anesthesia, n (%)	TURBT		2 (22.2)				
	Spinal		9 (100.0)				
Level of anesthesia, n (%)	General		0 (0.0)				
	T10		4 (44.4)				
	T8 Post		5 (55.6)				
Onset of erection	urethroscope Pre		7 (77.8)				
	urethroscope		2 (22.2)				
Ketamine total dose (mg)	Mean (SD)		34.3 (19.5)				
	Median (min-max)		25.0 (18.0–75.0)				
Duration of operation (min)	Mean (SD)		91.7 (31.3)				
	Median (min-max)		90.0 (40.0–140.0)				
Outcome, n (%)	Resolved-delayed procedure		7 (77.8)				
	Conversion to general anesthesia		2 (22.2)				

*ASA* American Society of Anesthesiologists, *TURP* Transurethral resection of the prostate, *TURBT* transurethral resection of bladder tumor

The mean (SD) age was 68.3 years (range, 66.0–77.0 years). ASA physical status category I 6 7 and II were noted in 55.6 and 44.4% of patients, respectively. All cases received spinal anesthesia (*n* = 9) at T8–10 dermatome levels, for TURP in 7 (77.8%) cases and for TURBT in 2 (22.2%) cases. The onset of penile erection was post-urethroscope in 7 (77.8%) cases. The average total ketamine dose was 34.3 mg (range, 18.0–75.0 mg) and the duration of the operation was mean 91.7 min (range, 40.0–140.0 min). Ketamine treatment resulted in resolved erection with delayed procedure in 7 (77.8%) cases, while conversion to general anesthesia was required in 2 (22.5%) cases (Table 2).

The cases with ketamine failure included a 74-year old patient undergoing TURP and a 77-year old patient undergoing TURBT and both were post-urethroscopic rigidities (Table 2).

## Discussion

The findings of this study revealed that of 402 endourology operations performed in our clinic over a 4-year period (2015–2019), 9 (2.2%) patients had a penile erection during endourological surgery with spinal anesthesia. Overall, 7 of 9 (77.8%) cases were successfully treated with intravenous ketamine (total dose ranged from 18.0 to 75.0 mg) administration, which resolved penile rigidity and the operation was completed within 91.7 min on average.

The overall prevalence of intraoperative penile erection under anesthesia has been reported to range from 0.1 to 2.4% [5], specifically at 0.34–3.5% for general anaesthesia, 0.11–0.3% for spinal anaesthesia and 1.72–3.8% for epidural anaesthesia [3–5]. Hence, the prevalence of intraoperative penile erection in the current cohort of endourology patients seems to be consistent with previously reported rates for endourology procedures performed under spinal anesthesia.

Although the exact mechanism has not been clarified, penile erection under anesthesia is considered to be mediated by both psychogenic and refloxogenic stimulation [4, 14], while the latter is considered to be more common via the stimulatory effect of washing, touching and 6 7 instrumentation of the genital area on sacral root afferents [4]. Therefore, with the onset of penile erection post-urethroscope in 77.8% of the current study cases, these findings support the view that intraoperative penile erection generally follows local stimulation of the penis during skin preparation or instrumentation due to activation of sacral parasympathetic pathways eliciting an unopposed reflex response via an autonomic imbalance [3–5]. In addition, all of the intraoperative penile erection cases in this cohort had received spinal anesthesia with T8-T10 blockage, in accordance with the higher prevalence of erections with blocks reaching higher than T8 and rarely with those lower than T12 [16].

Notably, while the condition has been considered to occur predominantly in younger males [3–5], in the current cohort, the median age of patients with intraoperative penile erection was 69.0 years (range, 60.0–77.0 years), with the potential likelihood of treatment failure in older patients.

These findings demonstrated that the intravenous injection of ketamine was an effective and safe method for immediate relief of intraoperative penile erection with a high success rate. This supports the reported efficacy of ketamine, as a dissociative anesthetic, in the management of intraoperative penile erection in past studies [2, 6, 11–13].

Moreover, in 7 of 9 cases, ketamine administration resolved penile rigidity enabling completion of the operation within an average of 91.7 min with no side-effects. This seems notable given the consideration of prolonged time to achieve flaccidity (range, 90 min^− 2^ h) being the major disadvantage of intravenous ketamine treatment, together with the potential risk of hallucinations and unpleasant dreams in patients under concomitant spinal anesthesia [4, 11, 17].

Certain limitations to this study should be considered. First, due to the retrospective single 1 2 3 4 5 center design of the study, establishing the temporality between cause and effect seems difficult. Second, generalizing these findings to the whole endourology population is not 6 7 possible due to the relatively low sample size. Nevertheless, despite these certain limitations, given the paucity of solid information available in accordance with the rarity of the condition and the challenges regarding conduction of very large-scale studies to determine the benefits of any therapeutic intervention, these findings represent a valuable contribution to the literature.

## Conclusion

In conclusion, the prevalence of intraoperative penile erection during spinal anesthesia for endourological surgery was 2.2% in our experience. These findings demonstrated that intravenous injection of ketamine is an effective and safe method for immediate resolution of intraoperative penile erection with a high success rate.

## Data Availability

The datasets used and/or analysed during the current study are available from the 1 2 3 4 5 corresponding author on reasonable request.

## Abbreviations

ASA: American society of anesthesiologists
TURBT: Transurethral resection of bladder tumor
TURP: Transurethral resection of prostate
